# Visual Quality, Motility Behavior, and Retinal Changes Associated with Reading Tasks Performed on Electronic Devices

**DOI:** 10.3390/life13081777

**Published:** 2023-08-20

**Authors:** Elvira Orduna-Hospital, María Munarriz-Escribano, Ana Sanchez-Cano

**Affiliations:** Department of Applied Physics, University of Zaragoza, 50009 Zaragoza, Spain; mariamunarriz00@gmail.com

**Keywords:** eye tracker, eye movements, aberrometry, visual quality, optical coherence tomography

## Abstract

Background: The purpose of this study was to objectively evaluate visual discomfort using an eye tracker and aberrometer after a 21-min reading session on an iPad and an Ebook. Additionally, retinal changes were analyzed using optical coherence tomography (OCT). Methods: A total of 31 young subjects (24 ± 4 years) participated in this study. They read for 21 min on an Ebook and for another 21 min on an iPad under controlled lighting conditions while their eye movements were monitored using an eye tracker. Aberrometry and retinal OCT measurements were taken before and after each reading session. Parameters such as pupil diameter, fixations, saccades, blinks, total aberration, high-order aberration, low-order aberration, and central and peripheral retinal thickness in the nine early treatment diabetic retinopathy study (ETDRS) areas were measured for each reading situation. Statistical analysis was performed on the collected data. Results: No statistically significant differences (*p* > 0.05) between the two devices were observed in terms of the different types of eye movements or the changes in retinal thickness. However, the aberrometric analysis showed variations in post-reading situations depending on the device used. Conclusion: Reading speed and visual discomfort resulting from electronic device usage can be objectively assessed using an eye tracker and aberrometer. Additionally, changes found in central and peripheral retinal thickness between the two devices and the baseline measurements were not significant and remained relatively stable.

## 1. Introduction

Recently, the use of near vision (NV) has been steadily increasing for the execution of various tasks, whether they involve work, study, or entertainment. Activities such as reading, performing tasks, and viewing videos or movies on digital platforms are increasingly being carried out using electronic devices (computers, tablets, or smartphones).

The consequences of the continuous and prolonged use of these devices result in various ocular symptoms, such as irritation, dryness, itching, tearing, burning, or a foreign body sensation [1,2], leading to visual fatigue, diplopia, blurred vision, or asthenopia, among other conditions. These signs mainly appear in individuals who spend four or more consecutive hours using these devices, with symptoms intensifying with daily use exceeding seven hours [3,4], affecting the attention and performance of individuals who experience them; this condition is known as computer vision syndrome (CVS) [5]. It is estimated that the prevalence of CVS can range from 25% to 93%, depending on the device used, the duration of use, and environmental factors. The number of people affected is projected to increase significantly each year [3,4].

Objective signs also appear, such as changes in accommodation and optical manifestations such as ocular aberrations in response to prolonged NV work [6,7]. Wick and Morse demonstrated that the accommodative response (accommodative lag) increases in computer work [8]. However, Moulakaki et al. argued that the accommodative response is independent of the device used [9]. Other studies show that both low-order aberrations (LOAs) and high-order aberrations (HOAs) increase with accommodation, particularly spherical aberrations. These changes may be due to alterations in the different ocular media during the accommodation process, resulting in an imperfect image formed on the retina [10]. Changes in retinal thickness and shape have also been observed during reading under different lighting conditions, especially in low light conditions in multiple macular areas [11].

CVS is a prevalent condition that can cause discomfort and affect productivity and quality of life; there is no universally agreed-upon definition or diagnostic criteria, and the best interventions are not well established [2]. It is known that computer use reduces the frequency and amplitude of blinking; however, for handheld devices such as tablets, e-books, mobile phones, etc., this has not been conclusively proven, although it is assumed that they affect tear stability. Despite this finding, there are no irrefutable studies to confirm that discomfort or other symptoms such as asthenopia caused by device use is related to blinking [12].

There are two main types of e-readers: e-books, which are designed to resemble printed books on paper, and liquid crystal display (LCD) screens, which are used in most electronic devices such as iPads or tablets. Reading on an LCD display can cause more visual fatigue than reading a paper book or E-ink display, likely due to the higher level of luminance emitted by the LCD display, which can cause the pupil to constrict and the frequency of eye blinks to decrease. These changes can lead to eye strain and fatigue [13]. In spite of this, one advantage of using these technologies is the ability to customize factors such as font type and size, screen luminance, and even background color for reading, allowing for individualized adaptation to the user’s preferences [13].

Reading speed is a measure of how quickly a person can read and comprehend text. It is an important indicator of reading fluency, which is essential for academic success, as it allows students to read and understand complex texts in a timely manner. In general, reading speed increases with age and may be affected by visual discomfort, which is caused by several factors, including reading for long periods of time, reading in poor lighting conditions, or reading a text that is too small or too difficult [14].

Some studies have focused on how ambient lighting, contrast, and the difference in luminance between the background and text affect visual comfort when reading on electronic devices [15]. Others have specifically investigated the optimal ambient lighting conditions. It is specified that the minimum illumination should be 200 lux for comfortable and pleasant reading [16]. If the ambient lighting is too high, the visibility of the screens may be compromised, contrast can be lost, and reading can become more challenging. However, for E-ink devices without backlighting, the ambient lighting needs to be even higher, preferably above 700 lux, to ensure effective readability [17].

The reading process involves saccades, which are small and quick jumps lasting approximately 20–40 ms with which the gaze direction is changed, causing the image of the object of interest to remain on the fovea. These are binocular movements, where both eyes (OU) move in the same direction, performing conjugated movements (versions) following the line of the text being read [18,19]. Following a saccade, a fixation occurs, which is the time in which the gaze remains on the object of interest for approximately 200 to 250 ms; these are the moments in which reading is carried out [19]. Fixations do not occur word by word but rather involve reading groups of words, valued as reading efficiency [20]. The concept of “regression” refers to fixations that occur from right to left, backward movements in reading to reread a word or group of words, and movement to the next line [21].

Generally, proficient readers employ a lower number of fixations and spend less time on them compared to less skilled readers. The latter group experiences more difficulty comprehending the text as they analyze the meaning of each word rather than taking a global approach to the context [21]. Many individuals struggle to comprehend what they read or find that they require a significant amount of time to read and understand, leading to frustration and inhibiting skill development. This highlights the importance of emphasizing reading fluency and comprehension at an early age [22].

The eye tracker is an electronic device that records gaze tracking, allowing for the measurement of unconscious eye movements during specific tasks. It provides insights into the skills and cognitive processes of the subject being evaluated. Eye tracking is a scientific research method used in various fields, including advertising [23], psychology [24], human–computer interaction [25], and some optometric tests [26,27,28]. The gaze-mind hypothesis is investigated by examining individuals’ preferences based on the direction of their visual axes [29].

The primary objective of this study was to assess reading speed, monitored with an eye tracker, to evaluate visual discomfort, aberrometric changes, and retinal thickness in different quadrants after sustained NV tasks using an iPad or e-book under controlled lighting conditions.

## 2. Materials and Methods

### 2.1. Sample Description and Selection

The study was conducted in accordance with the principles outlined in the Declaration of Helsinki and with the approval of the Clinical Research Ethics Committee of Aragón (CEICA) under reference number PI21-074 and with signed informed consent from the participants. The sample consisted of 31 healthy individuals, 20 females and 11 males, with an age range between 18 and 31 years. A comprehensive optometric evaluation was performed on the participants, including measurement of the best-corrected visual acuity (BCVA) in both distance vision (DV) and NV, monocular accommodative amplitude, accommodative and convergence facility in both monocular and binocular vision, associated and dissociated phoria measurement, positive and negative fusional vergences at near and distance, and fusion and stereopsis; ocular motility was also assessed. Participants who required optical correction were asked to bring their contact lenses since the antireflective coating is designed for the wavelength range of 400–700 nm; it also reflects other wavelengths, including the infrared used by the eye tracker (>750 nm). Subjects who met the exclusion criteria were not able to participate in the experiment. These criteria included binocular vision problems, BCVA less than 0.8 decimal in one of the eyes, vision-impairing pathologies, media opacities, dry eye syndrome, use of electronic devices within one hour before the measurements, consumption of coffee, smoking, engaging in high-intensity exercise, or attending the session without their contact lenses corrected for DV since they were young participants and had enough accommodation to focus on NV.

### 2.2. Devices Used, Setup, and Lighting

One of the instruments used for the measurements in the study was the Tobii Pro Fusion Eye Tracker (Tobii AB, Danderyd, Sweden). This device operates by emitting infrared light at around 850 nm that produces a corneal reflection captured by a camera within the device. By analyzing the corneal reflection and pupil position, the device determines the direction of the visual axes and estimates where the subject is looking. Prior to reading, individual calibration is performed for each person by directing their gaze to calibration points displayed on the screen to be used, providing information about the duration of saccades and fixations [30].

Two different electronic devices were used for reading: an 8th generation iPad, Model A2270 (Apple Inc., Cupertino, CA, USA), with screen dimensions of 250.6 × 174 × 7.5 mm and 2160 × 1620 pixels and an E-ink reader Ebook (ink pad 3, PocketBook International, Lugano, Switzerland), model PB740, with dimensions of 195 × 136.5 × 8 mm and 1872 × 1404 pixels. Times New Roman font was used with a size of 9 pixels for the iPad and 10 pixels for the Ebook (slightly different by the resolution described for each screen). Thus, visual acuity was achieved of around 0.8 decimal in both devices when reading at 50 cm.

During the reading sessions, recordings were made, requiring a camera with a microphone connected to the laptop from which the readings were monitored using eye tracker software. The exact camera model used was AMDIS01B (Conceptronic, Dortmund, Germany), while the software programs employed were the Eye tracker Manager (Tobii AB, Danderyd, Sweden) for selecting the device used for reading and the Tobii Pro Lab (Tobii AB, Danderyd, Sweden) for individual calibration of each subject for each reading session.

**Figure 1:**
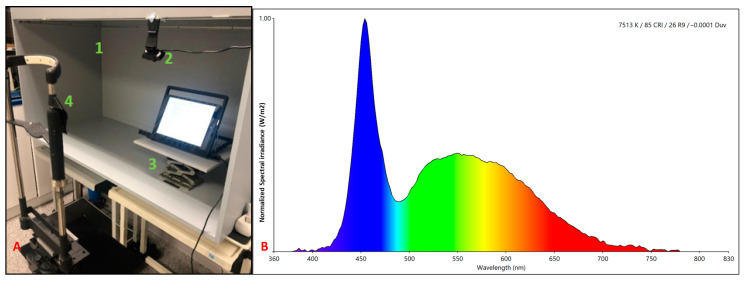
Image (**A**) Study elements. (1) Light control cabinet. (2) Camera. (3) Stand for device and eye tracker support. (4) Chin rest. Image (**B**) Normalized spectral irradiance (W/m^2^) of the ambient light reaching the corneal plane while reading.

The experimental components were placed within a light control cabinet. In addition, two additional tools were used for the measurements: a chin rest and a stand to hold the reading device (Figure 1).

The experiment consisted of two readings: one using an iPad and the second using an Ebook, randomly assigned, with both readings conducted under controlled lighting conditions. To determine the irradiance (W/m^2^) and illuminance (lux) on the corneal plane and the luminance (cd/m^2^) of the reading devices, a spectroradiometer (model StellarNet-Black Comet, StellarNet, Inc., Tampa, FL, USA with C20080502 calibration and NIST traceability) and a luminance meter (Mavo-Spot 2, Gossen-Kainos, Barcelona, Spain) were used. The luminance perceived by the eye and emitted by the iPad was 59.57 cd/m^2^ and 58.01 cd/m^2^ for the Ebook. The illuminance reaching the corneal plane was 257.0 lux for both the iPad and the Ebook and the irradiance was 0.91 (W/m^2^) for the iPad and 0.87 (W/m^2^) for the Ebook, as shown in Figure 1.

The analysis of visual quality was performed using the IRX3 Hartmann–Shack aberrometer (Image Eyes, Orsay, France). This device uses a light source of 780 nm that is projected onto the retina and, based on the impact of the rays coming from it on a CCD, generates a map of the total aberration of the evaluated eye.

The study of retinal changes was carried out by capturing images of multiple retinal layers using the 3D OCT-1000 model (Topcon Corporation, Tokyo, Japan). The light source was a superluminescent diode with a wavelength of 840 nm and a bandwidth of 50 nm. The longitudinal (depth) resolution was 6 µm (A-scan) and the maximum transverse (horizontal) resolution was 20 µm (B-scan).

**Figure 2:**
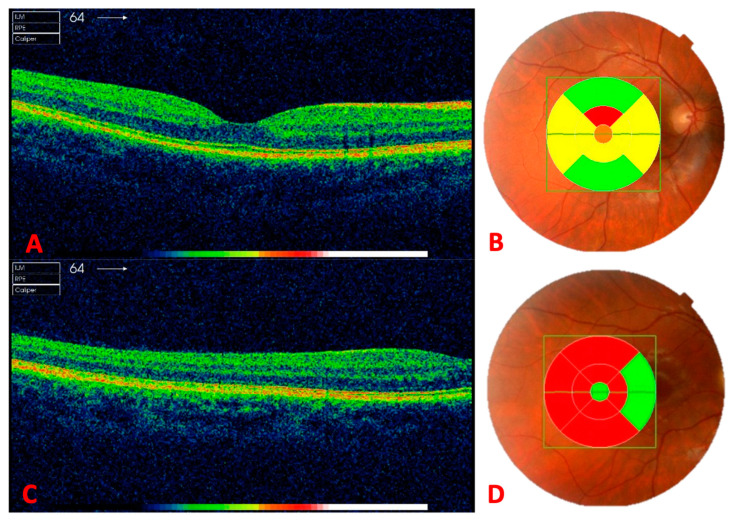
Image (**A**) A tomographic cross-section of the central retina. Image (**B**) The fundus of the eye with central retinal thickness in the 9 quadrants of the early treatment diabetic retinopathy study (ETDRS). Image (**C**) A tomographic cross-section of the peripheral retina. Image (**D**) The fundus of the eye with peripheral retinal thickness in the 9 quadrants of the ETDRS. All these images correspond to the right eye (OD) of the same individual.

The protocol followed with each participant involved obtaining baseline measurements of OU separately using the aberrometer and four measurements using OCT, two for each eye, in a random order each time, always under scotopic lighting conditions. The participant was asked to focus on the central square that appeared, thus capturing the image of the central 30° of the retina centered on the fovea. The macular cube protocol was performed, capturing 128 tomographic retinal slices, with the macula in the center of the image (Figure 2A,B). The second captured image involved looking toward the temporal end of the central line, thus obtaining 128 baseline images of the temporal peripheral retina using the same macular cube protocol but with the eye rotated 15° toward the temporal side (Figure 2C,D).

The proposed two readings were performed 50 cm from the reading device using either the Ebook or the iPad. The eye tracker was calibrated for each subject and each reading device, ensuring that the eye tracker detected OU. A calibration template consisting of numbers from 1 to 5 was used. The subject was instructed to sequentially fixate on each number until all of them had been completed. From the computer, the examiner could observe which point the subject was looking at and accept or reject the calibration.

**Figure 3:**
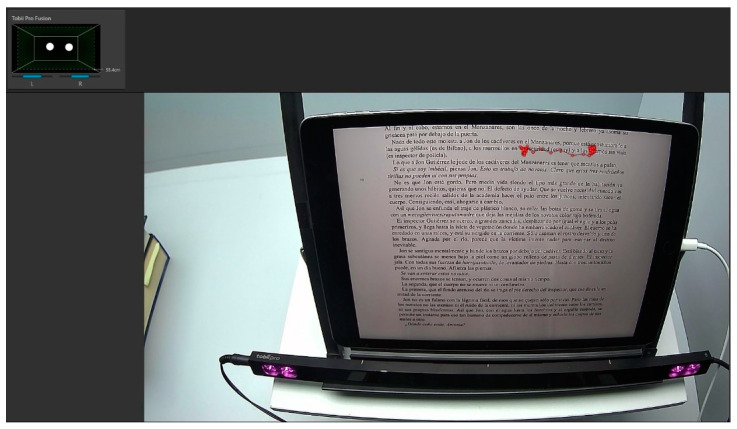
The upper left quadrant indicates the detection of both eyes (OU) of the subject. The red line observed over the text of the reading device (iPad) represents eye movements, tracking the line of text during reading.

The reading session lasted for 21 min. The examiner recorded one minute of reading every 5 min to evaluate the reading process and assess the eye movements and visual discomfort throughout the reading session (Figure 3). This resulted in a total of five-1 min recordings.

Immediately after the 21 min reading session, the same baseline tests were repeated with an aberrometer and OCT. The experiments were performed in the morning and the participants were instructed to take a 15 min break, emphasizing the need to maintain the same conditions as 1 hour before the tests. For the second reading session, the same procedure was followed, but this time, the device that was not used in the first reading session was employed.

### 2.3. Data Export and Statistical Analysis

**Figure 4:**
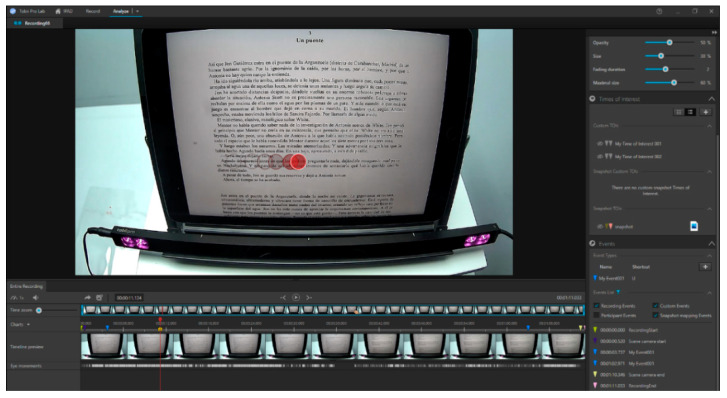
Segmentation of the reading recording with the iPad using Tobii Pro Lab. The blue markers indicate the start and end of a 1 min segment of the recording. The red circles indicate the eye movements made during the reading session.

**Figure 5:**
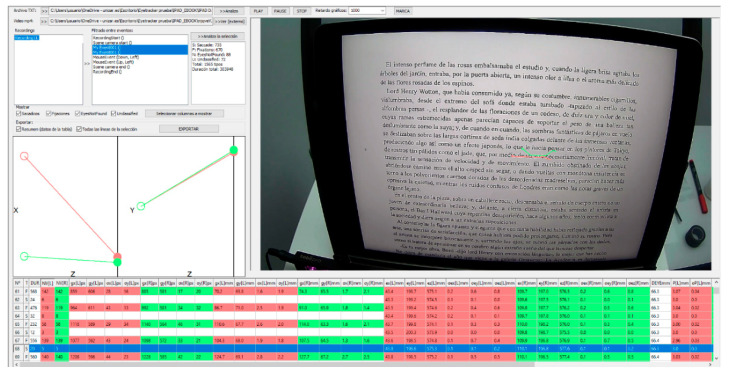
Analysis of a selected event from a reading session with the iPad using the Etracker Parse program. The lines overlaid on the text represent a saccade, which indicates rapid eye movement during the reading process.

The data collected with the aberrometer were exported to an Excel database (Microsoft^®^ Office Excel 2011, Microsoft Corporation, Redmond, WA, USA). The recordings taken with the eye tracker were segmented using Tobii Pro Lab software. The “events” option was used to select the start of reading by the subject and, after 60 s, the segment was cut using the same option. Two markers appeared in the recording to delimit the segment (Figure 4). This process resulted in a total of 5 min divided into 5 different 1 min recordings. After performing the same process for all participants, each recording was individually exported in Excel format. For the analysis of the exported data, a program called Etracker Parse Video (University of Zaragoza, Zaragoza, Spain) was created (Figure 5). This program allowed for the selection of the data of interest during the “events,” such as the total duration (s), number (n) of blinks, saccades, fixations, pupil diameter of the left eye (oculus sinister, OS) and right eye (oculus dexter, OD) (mm), length (mm), duration (ms), and velocity (m/s) of the saccades for each eye separately, as well as the average duration of fixations (ms).

The analysis of all collected data was conducted using the Statistical Package for the Social Sciences (SPSS) version 24.0 (IBM Corp., Armonk, NY, USA). Descriptive statistics were initially performed on the quantitative variables, including the calculation of the mean, standard deviation (±SD), maximum, and minimum values. The normality distribution of all variables was assessed using the Kolmogorov-Smirnov test, which indicated that the sample did not follow a normal distribution. Therefore, nonparametric tests for related samples, specifically the Wilcoxon signed-rank test, were employed to examine differences between the variables when comparing the two reading conditions and, in the case of aberrometry and OCT measurements, comparing them with the baseline measurements. A value of *p* < 0.05 was considered to indicate statistical significance.

## 3. Results

Thirty-one young subjects (mean age: 24 ± 4 years) who met the inclusion and exclusion criteria described in the previous sections were selected.

### 3.1. Eye Tracker

**Figure 6:**
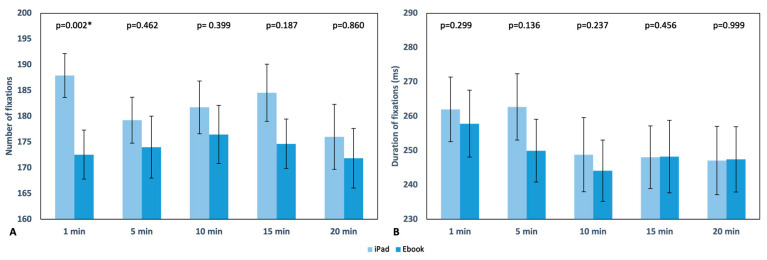
Graphical representation of the number (**A**) and duration (ms) (**B**) of fixations in each reading recording with the iPad and Ebook, respectively. The upper area of the graph displays the *p* value, indicating * statistically significant differences; standard error is also displayed as error bars.

Fixations. The number of fixations during reading with the iPad reached its maximum value in the first minute of reading (187.90 ± 23.59), while with the Ebook, it occurred at 10 min (176.45 ± 31.47). In both devices, the minimum number of fixations was observed at the 20th minute, with 176 ± 35.10 and 171.84 ± 32.25 fixations, respectively. However, statistically significant differences were found only at the beginning of the test (*p* = 0.002), as shown in Figure 6A. There were no statistically significant differences in the duration of fixations during the readings (Figure 6B), although, during the first 5 min, these fixations had a longer duration compared to the subsequent time intervals recorded. Overall, it can be observed that the use of the iPad, compared to the Ebook, led to a higher number of fixations on the text during reading, as well as longer fixation durations.

**Figure 7:**
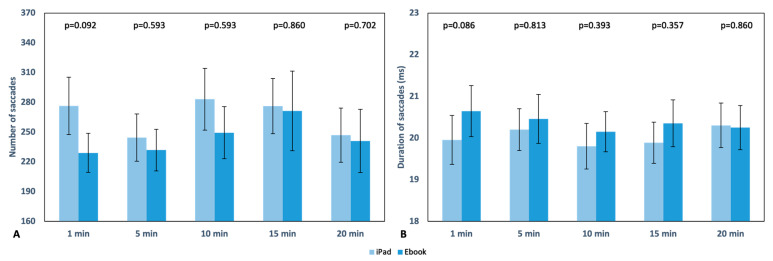
Graphical representation of the number (**A**) and duration (ms) (**B**) of saccades in each reading recording with the iPad and Ebook, respectively. The upper part of the graph displays the *p* value; error bars are also displayed.

Saccades. During reading with the iPad, the maximum number of saccades occurred at the 10th minute (283.13 ± 173.29), while with the Ebook, it was at the 15th minute (271.39 ± 223.51). The minimum number of saccades was observed in the 5th minute (244.42 ± 132.92) for the iPad and in the first minute (229.03 ± 109.82) for the Ebook. In this case, no statistically significant differences were found in the duration or number of saccades (Figure 7). There were no differences observed throughout the reading; they remained consistent in both readings.

**Figure 8:**
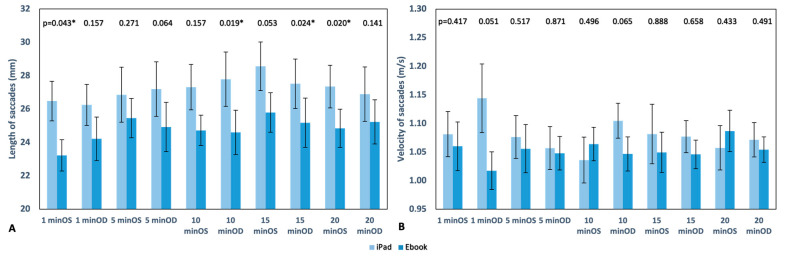
Graphical representation of the length (mm) (**A**) and velocity (m/s) (**B**) of saccades in each reading recording with the iPad and Ebook. The upper part of the graph displays the *p* value, indicating * statistically significant differences; standard error is also displayed as error bars.

In Figure 8, the length and velocity of saccades are shown separately for each eye (monocular); in general, both measures were higher in readings with the iPad, except for the 10th and 20th minutes with the OS, where velocity were higher with the Ebook. No statistically significant differences were found for velocity. However, for saccade length, significant differences were observed in the OS during the first minute and at the 20th minutes and in the OD at the 10th and 15th minutes, with *p* values of 0.043, 0.020, 0.019, and 0.024, respectively.

**Figure 9:**
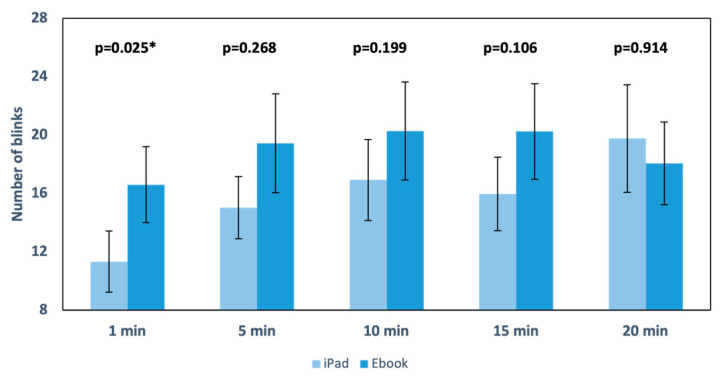
Graphical representation of the number of blinks during each recording in the two performed readings. The upper part of the graph displays the *p* value, indicating * statistically significant differences; standard error is also displayed as error bars.

Blinks. The number of blinks during reading with the iPad reached its maximum value at the 20th minute (19.76), while, with the Ebook, it was at the 10th minute (19.43). The minimum number of blinks for both devices occurred in the first minute and was 11.32 and 16.59 blinks, respectively. Significant differences were found only in the first minute of the test (*p* = 0.025) (Figure 9). In general, it can be observed that with Ebook reading compared to iPad reading, subjects blinked more, but, after 20 min of reading, the number of blinks tended to equalize.

Pupil. Throughout all the readings with each device, the pupil diameter remained constant for all subjects, with no significant changes in monocular analysis. When comparing the two reading instruments, significant differences were found at minutes 1 and 5 (*p* < 0.05). The largest recorded mean pupil size was 2.82 ± 0.39 mm in the first minute with the iPad, while the smallest recorded mean pupil size was 2.64 ± 0.31 mm with the Ebook, also during the first minute.

### 3.2. Aberrometry

**Figure 10:**
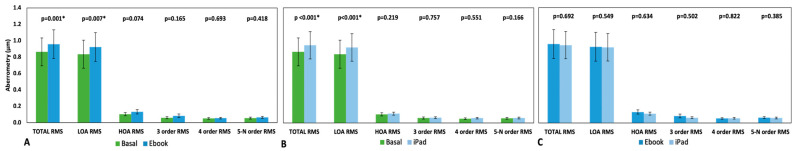
Comparative graphical representation of root mean square (RMS) (μm) before and after each reading (**A**,**B**) and by device after reading (**C**), considering OD and OS together. Low-order aberrations (LOAs), high-order aberrations (HOAs), 3rd-order aberrations RMS, 4th-order aberrations RMS, and 5th-order onwards (5-N) aberrations RMS. The *p* value is displayed above the graph bars, indicating * statistically significant differences; standard error is also displayed as error bars.

In Figure 10A,B, statistically significant differences (*p* < 0.05) can be observed when comparing the total root mean square (RMS_TOTAL_) between the baseline measurement and each of the reading devices separately, with an increase after reading. The statistical analysis indicated that differences were found in the low-order aberrations root mean square (RMS_LOA_) (*p* = 0.007 for the Ebook and *p* < 0.001 for the iPad), while the high-order aberrations root mean square (RMS_HOA_) did not show statistically significant differences in any case. When comparing both devices, no statistically significant differences were found as the *p* value was > 0.05 during all minutes of reading (Figure 10C).

### 3.3. Optical Coherence Tomography (OCT)

**Figure 11:**
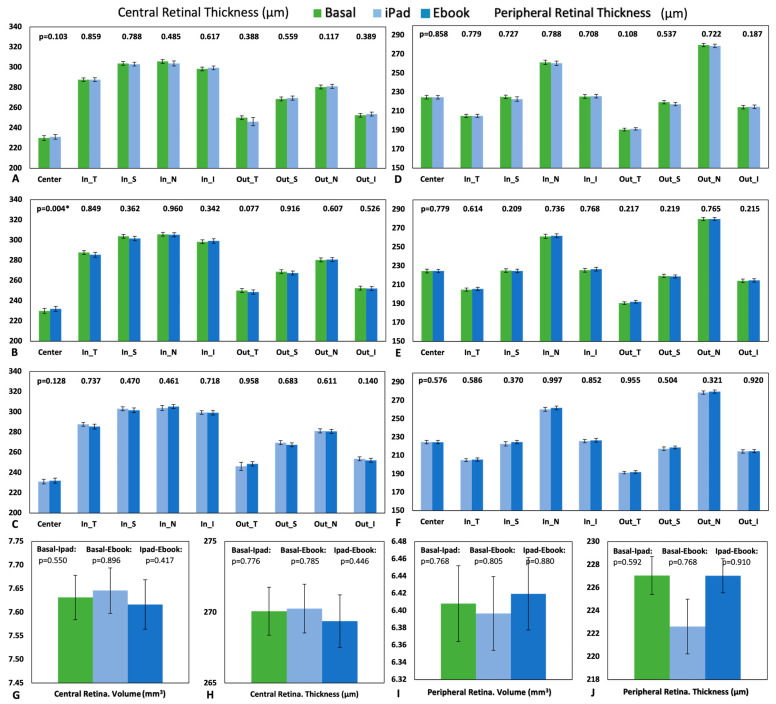
Graphical representation of the central retinal thickness measured by OCT: comparison of the baseline results with each device (**A**,**B**) and both devices with each other (**C**). Graphical representation of the peripheral retinal thickness measured by OCT: comparison of the baseline results with each of the devices (**D**,**E**) and between them (**F**). Graphical representation of the total central volume of the retina (**G**) and average total thickness (**H**) before the readings (baseline), after reading with the iPad, and after reading with the Ebook. Graphical representation of the total peripheral volume of the retina (**I**) and the average total thickness (**J**) before the readings (baseline), after reading with the iPad, and after reading with the Ebook. The *p* value is shown above the graphical bars, indicating * statistically significant differences; standard error is also displayed as error bars.

Central Retina. Significant retinal thickening (*p* = 0.004) compared to the baseline measurements was found when reading with the Ebook (Figure 11B) in the central ETDRS area. No statistically significant differences were found in Figure 11A,C, when comparing the basal retinal thickness and the retinal thickness after reading with the iPad and with Ebook, respectively. Figure 11G illustrates that there were no statistically significant differences (*p* > 0.05) in macular volume. The highest total volume in the central retina was observed after reading with the iPad, with a value of 7.65 ± 0.38 mm^3^, while the minimum volume was 7.62 ± 0.41 mm^3^ for the Ebook. In the comparison of average central retinal thickness between baseline measurements and post-reading measurements (Figure 11H), no statistically significant differences were found (*p* > 0.05), with the highest average thickness measured at 270.27 ± 13.54 μm with the iPad and the minimum average thickness at 269.38 ± 14.55 μm with the Ebook.

Peripheral Retina. No statistically significant changes were found in peripheral retinal thickness when comparing baseline measurements with post-reading measurements or between the two types of readings (*p* > 0.05) (Figure 11D–F). In Figure 11I, the highest total volume in the peripheral retina was observed after reading with the Ebook, with a value of 6.42 ± 0.33 mm^3^, while the minimum volume was 6.40 ± 0.33 mm^3^ for the iPad, with no statistically significant differences (*p* > 0.05). In the comparison of peripheral average retinal thickness between baseline measurements and post-reading measurements (Figure 11J), no statistically significant differences were found (*p* > 0.05), with the highest average thickness measured at 227.10 ± 13.10 μm in baseline measurements and the minimum average thickness at 222.62 ± 28.63 μm with the iPad, while the peripheral average retinal thickness after reading with the Ebook was 227.04 ± 11.69 μm.

## 4. Discussion

In this study, ocular motility was analyzed using an eye tracker through five one-minute recordings during a total reading time of 21 min using an Ebook and an iPad. Additionally, aberrometric changes were also studied to analyze the visual quality and modifications in retinal thickness after reading with each device, always under constant and comfortable ambient lighting conditions in the testing room.

Regarding fixations, as mentioned in the previous section, statistically significant differences were found between the two devices only in the average number of fixations during the first minute of reading (Figure 6A), with 187.90 ± 23.59 fixations for the iPad and 172.55 ± 26.57 fixations for the Ebook (*p* = 0.002). Overall, both the number and duration of fixations were higher during reading with the iPad. In the case of saccades, significant differences were found in their length (Figure 8A) (monocular analysis separately for each eye) in minutes 1 and 20 in the OS (*p* = 0.043 and *p* = 0.020, respectively) and in the OD at minutes 10 and 15 (*p* = 0.019 and *p* = 0.024, respectively), which could be explained by the subjective discomfort referenced by the subjects involved in our study. In contrast with our results, it has been described that parameters such as different screen refresh rates or distinct resolution do not affect saccadic eye movements or reading speed and accuracy [31,32]; this matches the results found for all other parameters such as number, duration, and velocity as there were no significant changes between the reading devices (Figure 7 and Figure 8B). 

Lighting conditions are crucial in these types of experiments. It has been observed that when both the luminance levels of the screens and the ambient lighting conditions are minimal, the total number of saccades and their duration, together with the number of blinks, are higher, which implies greater visual discomfort [26]. Poor ambient lighting conditions, characterized by uneven brightness between the screen and its background or reflections from the digital device can lead to discomfort and disabling glare, resulting in reduced contrast and a subpar image quality [33]. This diminished visual quality of electronic screens has been linked to a decrease in blink rates [34,35].

Regarding the blink rate (Figure 9), an increase in the number of blinks was observed throughout the reading with the Ebook, except at minute 20. The only significant value was at minute 1 (*p* = 0.025), indicating more blinks during reading with the Ebook, which is consistent with reading from printed paper, supporting the idea that backlit screens tend to decrease the blink rate (iPad) [36,37] and blinking abnormalities associated with changes in the ocular surface [2].

Expected values for the pupil diameter were obtained with both devices under levels of approximately 250 lux at the corneal plane. As intended, the pupil size remained constant in the monocular analysis. When comparing the two reading instruments, significant differences were found at minutes 1 and 5 (*p* < 0.05), perhaps due to the transient adaptation required to perform the experiment.

The relationship between blink frequency and tear film instability has been studied in previous research [36,37], which found that a lower blink frequency was associated with increased ocular dryness and tear film instability. Benedetto et al. [38] reported that an increased [13] blink frequency resulted in reduced tear evaporation, leading to improved tear film stability. According to Li et al. [39], not only did the subjective tear film stability increase but a higher number of saccades per second was also recorded. Although this was not observed in our study, a higher number of blinks was recorded during reading with the Ebook (Figure 9). Conversely, as observed in Figure 6A and Figure 7A, fixations and saccades were more frequent with the iPad, and the same trend was observed in fixations for their duration, as well as the length and velocity of the saccades. It was previously described that individuals experienced poor image quality, reduced contrast or font size, potential glare, or cognitive strain in relation to computer tasks and various testing conditions. Even when using handheld electronic devices at closer distances and below eye level, individuals reported lower blink rates, which may be attributed to the angle of gaze, although the exact cause remains unknown [40]. Talens-Estarelles et al. [41] found that the blink rate remained consistent across four different types of displays: computer, tablet, e-reader, and smartphone, suggesting that the blink rate may be influenced more by cognitive demands rather than the specific display method, as mentioned by other authors [42,43,44].

The analysis of the results obtained with the aberrometer indicates that, in both devices under the described illumination compared to baseline measurements, there is an increase in RMS_TOTAL_, specifically the RMS_LOA_, with statistically significant values in the comparison of baseline measurements with each device separately (*p* < 0.05). In both the iPad and the Ebook, the aberrations found were higher than those obtained in the baseline measurements. These results confirm a transient increase in RMS_LOA_, specifically defocus, after maintaining reading in electronic devices in young people with normal accommodation capacity.

The RMS_TOTAL_ after reading with the Ebook (Figure 10A) was 0.958 ± 1.374 μm and the RMS_LOA_ was 0.924 ± 1.376 μm, while, before the readings, they were 0.864 ± 1.340 μm and 0.836 ± 1.345 μm, respectively, with statistically significant differences (*p* = 0.001 and *p* = 0.007).

For the iPad (Figure 10B), after reading, the RMS_TOTAL_ was 0.945 ± 1.284 μm and the RMS_LOA_ was 0.918 ± 1.291 μm, while, before the readings, they were 0.864 ± 1.340 μm and 0.836 ± 1.345 μm, respectively, also with statistically significant differences (*p* < 0.001 in both cases).

However, no statistically significant differences were found for RMS_HOA_ in any of the comparisons. There were also no significant differences when comparing the values obtained between the iPad and the Ebook (Figure 10C).

There are many studies evaluating the anterior pole and digital devices [2], but the analysis of visual quality is still weak; comparisons in reading on paper versus reading on a computer screen under photopic conditions can be found [45]. In this case, after reading on the computer, individuals exhibited changes in aberrations below the 5th order. In the case of reading on paper, significant changes were observed in 3rd-order aberrations. When comparing both reading methods, no significant differences below the 5th order were found. In contrast, our study showed changes in LOA and total aberrations compared to baseline measurements. There were no statistically significant differences in aberrations between the two devices.

Regarding the retinal analysis, although the changes were not significant, it was found that when comparing central retinal thickness and volume (Figure 11G,H) and peripheral retinal thickness and volume (Figure 11I,J), the opposite phenomenon occurred. The highest thickness and volume in the central retina were observed after reading with the iPad (270.27 ± 13.54 μm and 7.65 ± 0.38 mm^3^, respectively), while the maximum thickness of the peripheral retina was found in the baseline measurement (227.10 ± 13.10 μm), and the maximum peripheral volume was obtained after reading with the Ebook (6.42 ± 0.33 mm^3^). When comparing peripheral retinal thickness after each reading (excluding the baseline measurement), the maximum thickness was found after reading with the Ebook. In other words, central thickening was observed after reading with the iPad, accompanied by peripheral thinning, while central thinning was observed after performing the task with the Ebook, resulting in peripheral thickening.

Although our results obtained with OCT are not statistically significant, they exhibit similar behavior in terms of central retinal thickness and volume compared to the aforementioned aberrometric results. According to another study in the literature [11], no significant differences were found in either central retinal volume or thickness; however, that study found differences in certain areas of the peripheral retina.

During reading, accommodation occurred, resulting in changes in ocular anatomy to achieve the necessary focus on the retina. This accommodation induced temporary myopia, and the data obtained in this study provide new insights into this topic. Electronic devices are considered a potential cause of myopia progression due to the sustained accommodation demand they require [46]. By maintaining maximum illumination and spatial frequency of the stimulus, axial defocus is sustained over time, stretching the retina, which is a myopiagenic factor [47], and even accommodative microfluctuations depend on the type of display used [48].

## 5. Conclusions

In conclusion, this study analyzed ocular motility and visual quality parameters during reading with Ebook and iPad devices. The findings demonstrated subtle variations in fixation and saccade patterns between the two devices, indicating a tendency towards increased numbers of fixations and saccades and longer durations of fixations during iPad reading, although in general not reaching statistical significance. Moreover, Ebook reading exhibited a higher blink frequency, potentially implying distinctions in tear film stability. The large errors associated with the measurement of eye motility make it difficult to say definitively whether the lack of statistical significance was due to the absence of the effect or the uncertainty of the measurements. Regarding visual quality evaluated by aberrometry, specifically, RMS_TOTAL_ and RMS_LOA_ increased significantly after reading with both devices compared to baseline measurements, suggesting a momentaneous myopization according to the sustained accommodation during reading; however, no significant differences were observed in RMS_HOA_.

Regarding retinal thickness, in our study population, no significant changes were found in central retinal thickness between the two devices, but a slight thinning was observed after Ebook reading compared to baseline measurements and peripheral retinal thickness remained relatively stable. These results, combined with the aberrometric results, suggest that the observed changes could be attributed to anterior pole changes during accommodation remaining in the retina with a certain stability after reading for 21 min under adequate lighting.

These findings contribute to the understanding of ocular responses during reading with electronic devices and highlight the importance of considering device-specific factors when assessing visual performance and ocular health. Nevertheless, further studies are needed to explore the long-term effects of using electronic devices for reading tasks and the potential implications of such use in myopia progression.

## Data Availability

Not applicable.
